# Respiratory Syncytial Virus Nonstructural Proteins Upregulate SOCS1 and SOCS3 in the Different Manner from Endogenous IFN Signaling

**DOI:** 10.1155/2015/738547

**Published:** 2015-10-18

**Authors:** Junwen Zheng, Pu Yang, Yan Tang, Zishu Pan, Dongchi Zhao

**Affiliations:** ^1^Department of Pediatrics, Zhongnan Hospital of Wuhan University, Donghu Road 169, Wuhan 430071, China; ^2^Department of Anatomy, School of Medicine, Wuhan University, Donghu Road 169, Wuhan 430071, China; ^3^Institute of Virology, College of Life Science, Wuhan University, Wuhan 430072, China

## Abstract

Respiratory syncytial virus (RSV) infection upregulates genes of the suppressor of cytokine signaling (SOCS) family, which utilize a feedback loop to inhibit type I interferon dependent antiviral signaling pathway. Here, we reconstituted RSV nonstructural (NS) protein expression plasmids (pNS1, pNS2, and pNS1/2) and tested whether NS1 or NS2 would trigger SOCS1 and SOCS3 protein expression. These NS proteins inhibited interferon- (IFN-) *α* signaling through a mechanism involving the induction of SOCS1 and SOCS3, which appeared to be different from autocrine IFN dependent. NS1 induced both SOCS1 and SOCS3 upregulation, while NS2 only induced SOCS1 expression. The induced expression of SOCS1 and SOCS3 preceded endogenous IFN-signaling activation and inhibited the IFN-inducible antiviral response as well as chemokine induction. Treatments with INF-*α* and NS proteins both induced SOCS1 expression; however, they had opposing effects on IFN-*α*-dependent antiviral gene expression. Our results indicate that NS1 and NS2, which induce the expression of SOCS1 or SOCS3, might represent an independent pathway of stimulating endogenous IFN signaling.

## 1. Introduction

Respiratory syncytial virus (RSV) is the ubiquitous cause of viral bronchiolitis and pneumonia in children younger than 1 year of age worldwide [1]. RSV is a single-stranded, negative-sense RNA virus belonging to the genus* Pneumovirus* and the family Paramyxoviridae [2]. The nonsegmented genome of RSV encodes 11 viral proteins, including two nonstructural (NS) proteins 1 and 2, which are unique features that distinguish the* Pneumovirus* genus from the rest of the Paramyxoviridae family [3]. The 3′ end locations of the* NS1* and* NS2* genes transcription might facilitate viral escape from the host's antiviral surveillance mechanisms [4, 5]. NS proteins are involved in the inhibition of the type I interferon (IFN) signaling pathway at various steps, including viral induction of IFN products and their signaling transduction, permitting viral replication [6–9]. Recombinant bovine RSV constructs lacking the* NS* genes and particularly lacking* NS2* are strong inducers of IFN-*α* and IFN-*β* expression in bovine nasal fibroblasts and bronchoalveolar macrophages [10].

When type I IFN binds to its receptor, this is the initial step in activating the Janus kinase (JAK) and signal transducers and activators of transcription (STAT) signaling pathway [11]. JAK/STAT activation results in the induction of IFN-dependent antiviral genes and of the suppression of cytokine signaling (SOCS) gene family members, a negative feedback loop for IFN signaling [12]. Traditionally, two pathways are considered to be involved in the activation of SOCS genes, one of which leads to the expression of cytokines such as IFN [13, 14]. In this way, SOCS proteins serve to balance the overshooting effect of cytokines. Viral genomic single-stranded RNAs and intermediate double-stranded (ds) RNAs are potent IFN modulators, serving as a ligand for pattern-recognition receptors (PRRs) in the regulation of the host's innate antiviral defenses [15, 16]. The genes encoding PRRs include Toll-like receptor 3 (*TLR3*) and retinoic acid inducible gene-I (*RIG-I*) [17, 18]. Single-stranded RNA viral genome or complementary dsRNAs can be recognized by the RIG-I and mitochondrial antiviral signaling (MAVS) pathways or by TLR3–TRAF6 receptors to activate nuclear transcription, promoting endogenous IFN expression, regulating the overly strong physiological effects of cytokines [19–23]. Another related mechanism involves viral proteins, such as influenza A NS protein and RSV G protein, which also upregulate SOCS expression, dependent or independent of the endogenous IFN pathway [24–26]. Both of these pathways for the upregulation of SOCS involve either endogenous IFN secretion or exogenous treatment. However, infection with RSV induces few endogenous IFN products because its NS proteins inhibit IFN signaling [27–29].

Because both the SOCS and NS proteins possess a demonstrated capacity to inhibit STAT phosphorylation, there is a possibility that NS protein expression is related to the upregulation of SOCS, inhibiting the STAT pathway prior to the endogenous activation of IFN signaling. Among the members of the SOCS family, the SOCS1 and SOCS3 proteins inhibit JAK enzymatic activity via Src Homology 2 recruitment to the receptor cytoplasmic domain, which results in the inhibition of JAK activity [30]. An additional level of regulation is provided by an E3 ubiquitin ligase complex that is bound to the SOCS box motif and ubiquitinates the associated proteins, targeting them for proteasomal degradation [28]. The RSV NS proteins can colocalize with MAVS and decrease levels of multiple members of the IFN pathways [31, 32], underscoring the importance of RSV NS proteins in regulating the antiviral immune defense.

In a previous study, we showed that RSV NS1 induced SOCS1 protein expression by inhibiting STAT2 phosphorylation [33]. Here, we investigated the different roles of NS1 and NS2 and both combined on SOCS expression and found that this regulation was a different way from interferon-alpha induction.

## 2. Materials and Methods

### 2.1. Cells and Viruses

Human A549 pulmonary epithelial cells were provided by the American Type Culture Collection (ATCC, Manassas, VA, USA) and grown in a 75 cm^2^ flask with Dulbecco's Modified Eagle's Medium containing 10% fetal bovine serum and 1% l-glutamine (with 100 U penicillin per mL and 100 U streptavidin pixels), incubated at 37°C in a 5% CO_2_/air incubator. The RSV A2 strain was from ATCC and was propagated and purified as described [34], snap-frozen, and stored at −80°C until use. The viral titer was determined by a standard plaque titration assay on the A549 cells.

### 2.2. Plasmid Construction and Transfection

Recombinant plasmids for RSV* NS1*,* NS2*, and coexpressing* NS1* and* NS2* (NS1/2) plasmids were designed according to the original* NS1* and* NS2* open reading frames (ORFs) of the wild-type RSV A2 strain (GenBank Accession number AF035006). However, the nucleotide sequences of the* NS1* and* NS2* genes were modified artificially for optimal expression in mammal host cells. The original* NS1* and* NS2* ORFs are unusually AT-rich; thus, by replacing the AT nucleotide pairs, we designed “humanized” sequences with redundant sequence structures containing regions rich in GC bases, which are more frequently used in mammalian host cell gene expression (*NS1* GenBank locus JQ900253.1 and* NS2* GenBank locus JQ900254.1; http://www.ncbi.nlm.nih.gov). Oligonucleotides covering the* NS1* and* NS2* ORFs without genetic information changes were synthesized (Invitrogen, Shanghai, China). Flag-tags and influenza hemagglutinin- (HA-) tags were added to the C′-terminals of the* NS1* and* NS2* ORFs. The synthesized sequences of* NS1* and* NS2* were subsequently cloned into the expression plasmid pcDNA3.1 (+) vector (Invitrogen, Carlsbad, CA, USA) and transformed into* Escherichia coli DH5-α* according to the manufacturer's instructions. To ensure efficient transcriptional termination of the inserted* NS1* and* NS2* genes, an internal ribosomal entry site sequence was inserted between* NS1* and* NS2* to coexpress the NS1 and NS2 proteins (pNS1/2), using Flag-tag and an HA-tag at the C′-terminals of* NS1* and* NS2*, respectively. The plasmid was transiently transfected with Lipofectamine 2000 (Invitrogen, Carlsbad, CA, USA), according to the manufacturer's instructions. In brief, cells were transfected with plasmids when growing as 60% confluence. For a 12-well plate, a mixture of 1.6 *μ*g of plasmid and 4 *μ*L of Lipofectamine 2000 was incubated in 200 *μ*L of Opti-MEM I Reduced Serum Medium (Opti-MEM; Gibco, Grand Island, NY) at room temperature for 20 min. The complexes were then added to each well containing cells and medium without antibiotics. The final plasmid concentration was 8 *μ*g/mL. The transfected cells were placed in a 5% CO_2_ incubator at 37°C for the indicated times.

### 2.3. Reverse Transcription Polymerase Chain Reaction (RT-PCR) and Real-Time Quantitative PCR (qPCR) Analysis

Total cellular RNA was isolated from the cells at various times after infection or transfection, according to the Trizol Reagent operation manual (Tri Reagent; Invitrogen, Carlsbad, CA, USA), and 1 *μ*g of RNA was reverse-transcribed in a 20 *μ*L reaction mixture using a M-MLV reverse transcription kit. qPCR was conducted using SYBR Green PCR Master Mix (Invitrogen, Carlsbad, CA, USA) according to the manufacturer's instructions. A 25 *μ*L aliquot of reaction mixture including 2 *μ*L of cDNA product, 12.5 *μ*L of SYBR Green SuperMix, and 10 *μ*M each of forward and reverse primers was amplified. The reactions were denatured for 2 min at 95°C and then run for 40 cycles of denaturation for 30 s at 95°C and annealed for 30 s at 65°C for human* MxA* gene forward 5′-GTTTCCGAAGTGGACATCGCA-3′ and reverse 5′-GAAGGGCAACTCCTGACAGT-3′, human* 2,5-OAS1* gene forward 5′-GATCTCAGAAATACCCCAGCCA-3′ and reverse 5′-AGCTACCTCGGAAGCACCTT-3′, human* SOCS1* gene forward 5′-TTGGAGGGAGCGGATGGGTGTAG-3′ and reverse 5′- AGAGGTAGGAGGTGCGAGTTCAGGTC-3-3′, and human glyceraldehyde-3-phosphate dehydrogenase (*GAPDH*) gene forward 5′-TGATGACATCAAGAAGGTGG-3′ and reverse 5′-TTACTCCTTGGAGGCCTAGT-3′.

The data were analyzed using a standard curve for each target gene generated by serial fivefold dilutions with the appropriate cDNA. The data were standardized against GAPDH and are presented as a relative value. For the delta/delta Ct method, the relative amount of target mRNA (2^−^ΔΔCt) was obtained by normalization to endogenous* GAPDH* reference gene expression.

### 2.4. Western Blot Analyses

To analyze whole cell lysates, cells were harvested at the indicated times, and protein extracts were prepared by adding a buffer containing 20 mM Tris-HCl (pH 7.4), 0.5% sodium deoxycholate, 10% glycerol, 150 mM NaCl, 2 mM EDTA, 50 mM *β*-glycerophosphate, 2 mM Na_3_VO_4_, 10 mM NaF, 1 mM DTT, 1 mM phenylmethylsulfonyl fluoride, and 0.1% protease inhibitor cocktail (Roche, Penzberg, Germany). The protein concentrations of the supernatant were determined using a protein assay kit (Beyotime Institute of Biotechnology, Shanghai, China). The sample was electrophoresed on a 5% stacking/10% separating sodium dodecyl sulfate polyacrylamide gel electrophoresis gel and transferred to a polyvinylidinedifluoride membrane (Immobilone; Millipore, Schwalbach, Germany). The membranes were blocked with 5% nonfat milk powder in 50 mM Tris-HCl (pH7.6), 0.15 M NaCl, and 0.1% Tween 20 for 1 h and then incubated with the following primary monoclonal (mAb) or polyclonal (pAb) antibodies. The primary antibodies were purchased from Cell Signaling Technology (Danvers, MA, USA) as follows: rabbit anti-human STAT1pAb; rabbit anti-human STAT2 pAb; rabbit anti-human phosphorylated STAT1 pAb; rabbit anti-human phosphorylated pSTAT2 pAb; rabbit anti-human SOCS1 pAb; rabbit anti-human SOCS3 pAb; rabbit anti-Flag mAb; and rabbit anti-HA mAb. Rabbit anti-human *β*-actin mAb was used as endogenous reference. The membranes were kept in dilution buffer with agitation at 4°C overnight and then washed and incubated for 2 h with peroxidase-conjugated goat anti-rabbit secondary antibody (Cell Signaling Technology). Protein bands were visualized using enhanced chemiluminescence plus Western blotting kits (Amersham Pharmacia Biotech, Buckinghamshire, UK) and imaged using Image Reader LAS-3000 (Fuji Photo Film, Tokyo, Japan). Then, the density of each band was measured using the ImageJ 1.46 program (NIH Image; http://imagej.nih.gov/ij/).

### 2.5. Enzyme-Linked Immunosorbent Assays (ELISAs)

Macrophage inflammatory protein- (MIP-) *α* (also chemokine (C-C motif) ligand 3 or CCL3), chemokine (C-C motif) ligand 5 (CCL5 or RANTES), and interleukin- (IL-) 6 concentrations were determined using ELISA kits purchased from Abcam Inc. (Cambridge, UK) with the appropriate matched antibodies according to manufacturer's instructions. Optical density at 450 nm was read on a Multiskan Ascent ELISA Reader (Thermo Labsystems, Helsinki, Finland).

### 2.6. Statistics

The results are expressed as the mean ± standard error (SE). Differences between means were analyzed using paired Student's *t*-tests and *p* < 0.05 was considered to be significant.

## 3. Results

### 3.1. Expression of Recombinant* NS1* and* NS2* Genes

**Figure 1:**
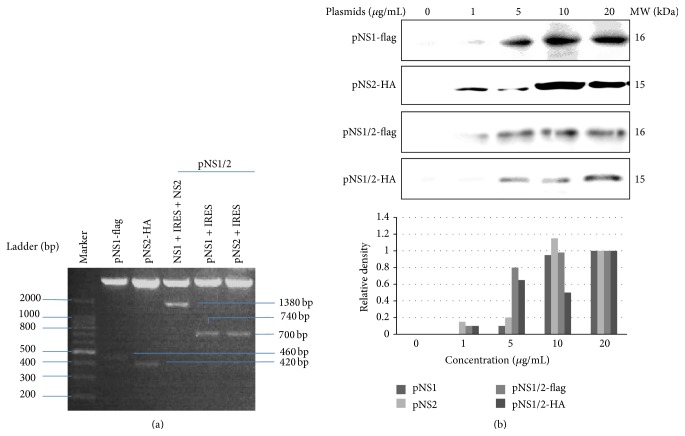
Identification and expression of the reconstituted plasmids pNS1, pNS2, and pNS1/2. (a) DNA gel electrophoresis analysis of the expression plasmids. pcDNA3.1(+) plasmids inserted with NS1, NS2, or NS1/NS2 artificial ORFs were digested with enzymes for 30 min, and the products were separated in a 1% agarose gel. (b) Total cellular extracts were collected from A549 cells at 2 h after plasmid transfection with final concentrations of 0, 1, 5, 10, or 20 *μ*g/mL, and Western blot was performed to measure protein levels. The data are shown as fold increases in protein density compared with the concentration of 20 *μ*g/mL, assumed to be 1.0.

After digestion with restriction endonucleases for 30 minutes, digested plasmid inserts were separated through electrophoresis on a 1% agarose gel, as shown in Figure 1(a). Three expected fragments were obtained: NS1-flag, NS2-HA, and NS1-flag-IRES-NS2-HA, with base sizes of 460 bp, 420 bp, and 1380 bp, respectively (Figure 1(a)). A series of concentrations of plasmids were transfected into A549 cells to detect the expression of target genes. The pcDNA(+) 3.1 vector was transfected into 60% confluent A549 cells, and cellular proteins were extracted for Western blot analysis. As shown in Figure 1(b), the expression of NS1-flag and NS2-HA as well as the coexpression of NS1-flag and NS2-HA could be detected in a concentration-dependent manner. The transfection concentration of 10 *μ*g/mL was shown to produce stable expression for all three plasmids and was selected for the following experiments.

### 3.2. NS1 and NS2 Induced SOCS1 and SOCS3 Expression in A549 Cells

**Figure 2:**
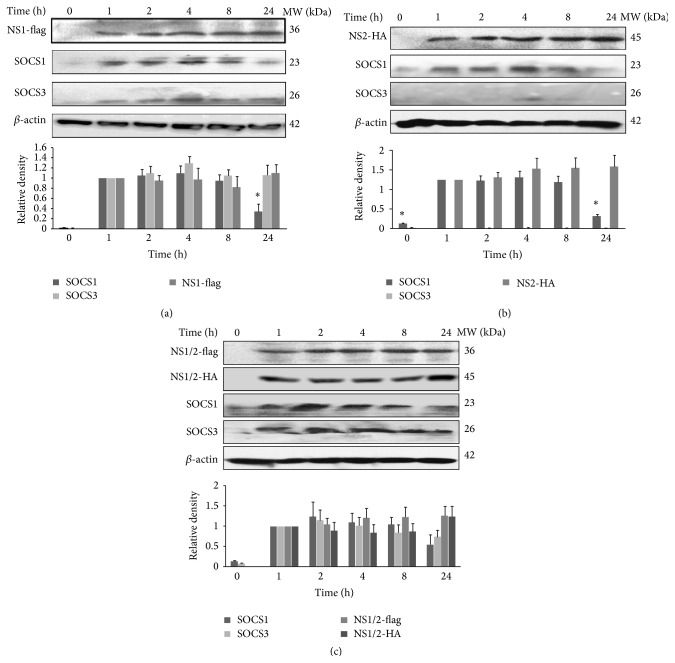
Nonstructural proteins NS1, NS2, and NS1/2 induced SOCS expression in A549 cells. A549 cells were transfected with NS1, NS2, NS1/2-expressing plasmids, or empty vector at final concentrations of 10 *μ*g/mL, and then total cell lysates were subjected to Western blot analysis using the following antibodies: anti-SOCS1 (1 : 500) and anti-SOCS3 (1 : 500) at the indicated times. (a) pNS1 transfection upregulated the expression levels of SOCS1 and SOCS3 proteins. (b) pNS2 transfection upregulated the expression levels of SOCS1 but SOCS3 proteins. (c) pNS1/2 transfection upregulated the expression levels of SOCS1 and SOCS3 proteins. The data were normalized to the level of *β*-actin and then to the same protein at time 1 h and are shown as fold increases in density compared with time 1 h from three independent experiments.

It is possible that the NS1 and NS2 proteins impair antiviral signaling in the early phase of infection. To clarify whether these NS proteins induced the expression of SOCS, A549 cells were transfected with pNS1, pNS2, or pNS1/2. The translated products of pNS1 and pNS2 were detected using Western blotting. NS1-flag fusion proteins were expressed stably. Significant increases in SOCS1 and SOCS3 protein levels were induced as soon as 1 h after plasmid transfection (Figure 2(a)). The same situation was found following pNS2 transfection, with the SOCS1 protein level increasing rapidly, but there was no effect on the SOCS3 protein level (Figure 2(b)). Coexpression of NS1/2 did not produce any synergetic effect on the expression of SOCS1 protein levels, but SOCS3 protein expression followed the same increased expression pattern as with pNS1 treatment (Figure 2(c)). The empty vector did not affect SOCS1 or SOCS3 expression levels. Based on these results, we conclude that the RSV NS1 and NS2 proteins are both key molecules in the induction of SOCS1 expression and that NS1 is mainly responsible for SOCS3 protein expression in the early phase of plasmid transfection.

### 3.3. NS1 and NS2 Displayed Different Roles in Impairing STAT Phosphorylation

**Figure 3:**
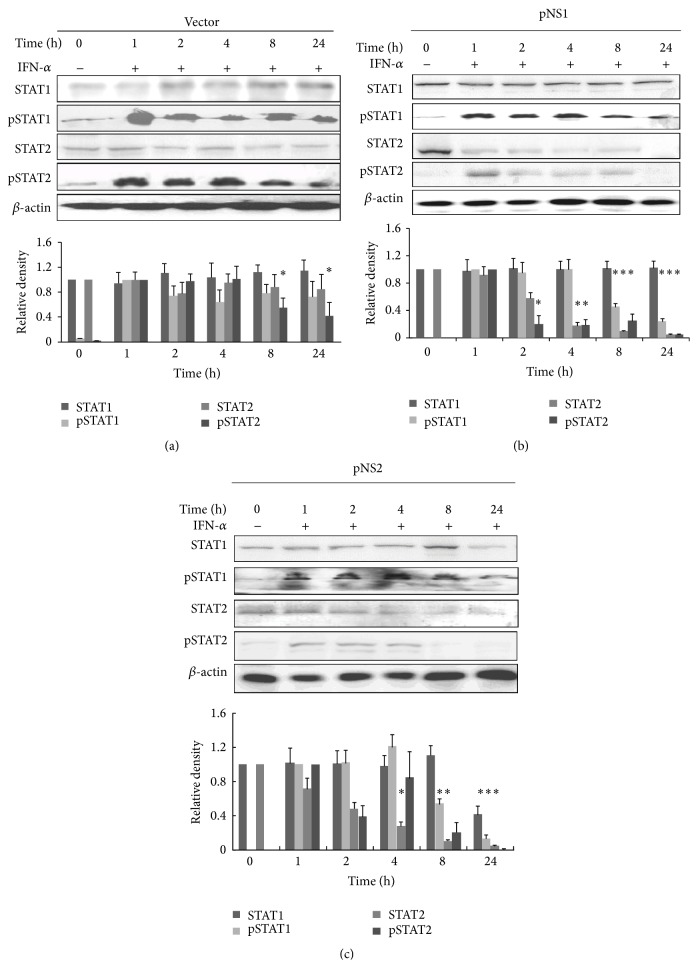
Expression of RSV NS proteins resulted in impaired IFN-*α*-induced STAT phosphorylation. A549 cells were incubated with or without IFN-*α* (5000 U/mL) for 30 min and then transfected with empty vectors (a), pNS1 (b), or pNS2 (c) for the indicated times. Total protein lysates were subjected to Western blot analysis using the following antibodies: anti-pSTAT1 (1 : 1000), anti-STAT1 (1 : 1000), anti-pSTAT2 (1 : 1000), or anti-STAT2 (1 : 1000). The results shown are representative of three independent experiments. The data were analyzed by densitometry, normalized to *β*-actin protein levels, and are shown as the mean ± SE. ^*∗*^ *p* < 0.05 versus untreated controls.

RSV is a weak inducer of IFN expression by binding the RIG-I protein to MAVS on the mitochondria and therefore blocks nuclear transcription and IFN production [35]. We had previously shown that NS1 upregulates SOCS1 expression independent of RIG-I or TLR3 [33]; in this study, the different roles of pNS1 and pNS2 on innate antiviral signaling mediated by SOCS1 and SOCS3 were detected. The early expression of SOCS could reduce STAT phosphorylation, which is related directly to viral protein functions. To clarify this regulation, the effects of NS proteins on STAT phosphorylation were determined. A549 cells were stimulated with IFN-*α* for 30 min and then transfected with pNS1, pNS2, or pNS1/2. The STAT phosphorylation was checked at the given time points. As shown in Figure 3(a), STAT1 and STAT2 phosphorylation increased after 1 h of IFN-*α* treatment and stayed at a high level throughout the observation period in vector transfected cells. pNS1 transfections suppressed IFN-*α*-inducible STAT1 phosphorylation at 8 h and STAT2 phosphorylation earlier at 2 h, diminished significantly over 24 h. In addition, pNS1 also degraded STAT2 at 4 h after transfection (Figure 3(b)). Treatment with pNS2 showed weak suppression of IFN-*α*-inducible STAT1 phosphorylation but blocked it completely by 24 h (Figure 3(c)) compared with NS1. The NS2 protein also demonstrated a stronger and earlier suppressive effect on STAT2 and its phosphorylation. NS1 and NS2 degraded STAT2 overtime while showing no effect on STAT1. Thus, RSV NS proteins probably play prominent role in regulating the phosphorylation of STATs, thereby acting as inhibitors of the type I IFN-induced JAK/STAT signaling pathway.

### 3.4. NS1 and NS2 Decreased IFN-*α*-Inducible 2,5-OAS1 and* MxA* Gene Expressions via SOCS1

**Figure 4:**
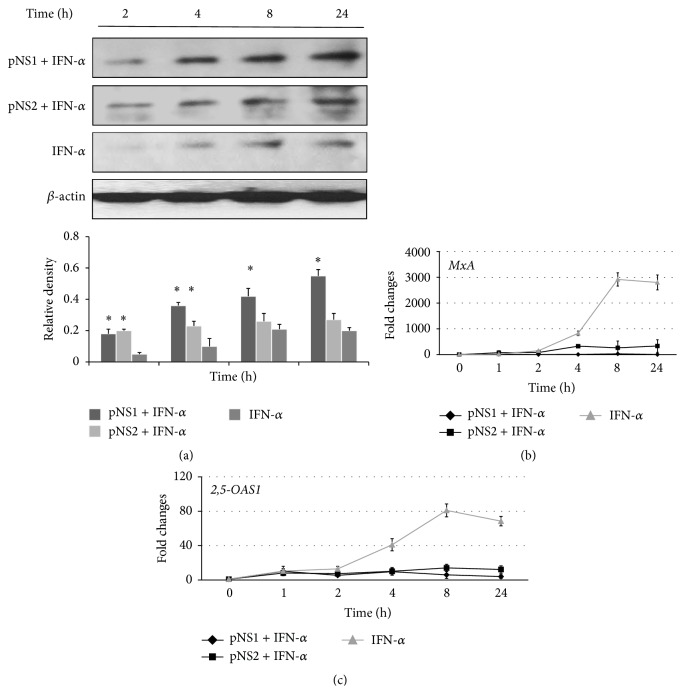
RSV NS proteins decreased the induction of IFN-*α*-induced antiviral genes through SOCS1 expression. A549 cells were treated with 5000 U/mL IFN-*α* for 30 min and were then transfected with pNS1 or pNS2 for the indicated times. Cellular total protein lysates were subjected to Western blot analysis of SOCS1 (a). The data were normalized to the level of *β*-actin and shown as the relative density from three independent experiments. *p* < 0.05 versus IFN-*α*. Total RNA was subjected to qPCR. The cDNA levels were analyzed using qPCR to assess the mRNA amounts of* MxA* or* 2,5-OAS1* (b, c). Equivalent mRNA amounts were normalized to* GAPDH* mRNA levels and calculated as *n*-fold changes compared with the levels of untreated cells at the respective time points. The results shown are representative of three independent experiments.

In view of the multiple functions of NS proteins, we analyzed how SOCS1 acted on IFN-induced antiviral gene expression. To further distinguish the overexpression of SOCS1 mediated through either IFN or NS proteins directly, A549 cells were treated with IFN-*α* and then transfected with pNS1, pNS2, or pNS1/2. Then, the mRNA expression levels from the IFN-*α*-dependent genes* 2,5-OAS1* and* MxA* were measured by qPCR at various time points. IFN-*α* stimulation led to a moderately elevated SOCS1 expression, similar to pNS2. Treatment with IFN-*α* and transfection with pNS1 or pNS2 showed a great stimulation of SOCS1 levels at 8 and 24 h (Figure 4(a)). pNS1 and pNS2 transfection induced SOCS1 expression within 1 h, whereas IFN-*α* treatment induced SOCS1 expression, but not until 4 h. The combined treatment yielded a biphasic curve in which the two effects were added together. These kinetics of SOCS1 expression patterns were quite different from those seen with plasmid transfections alone, in which the induced SOCS1 level peaked at an earlier phase (Figure 2(a)).

As expected,* MxA* and* 2,5-OAS1* mRNA levels increased significantly upon IFN-*α* treatment (Figures 4(b) and 4(c)). The expression of either NS1 or NS2 inhibited* MxA* and* 2,5-OAS1* mRNA transcriptions to near-background levels. These results indicate that the expression of NS1 or NS2 reduces IFN-*α*-mediated antiviral gene expression via upregulation of SOCS1 expression. Although INF-*α* and NS proteins both induced SOCS1 expression, they showed opposing effects on IFN-*α*-dependent antiviral gene induction.

### 3.5. RSV NS Proteins Suppressed the Expression of Proinflammatory Cytokines

**Figure 5:**
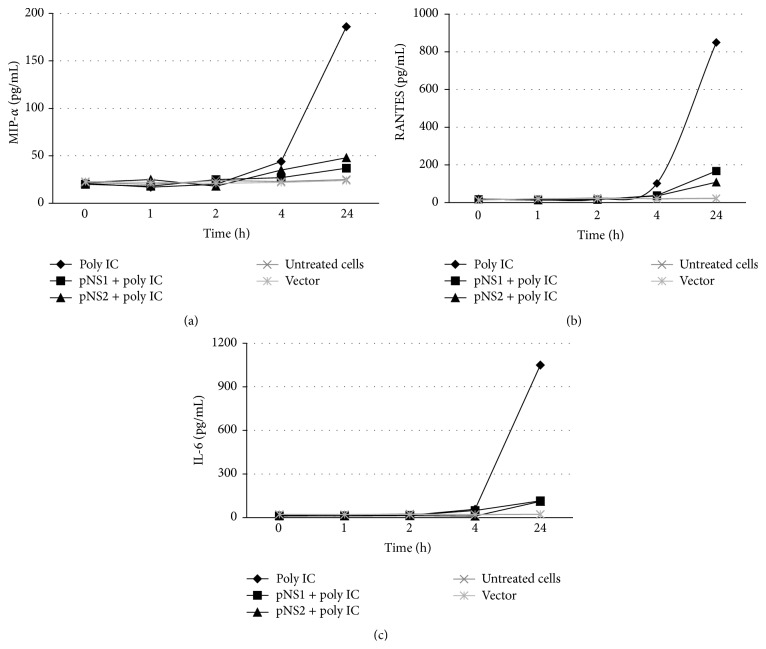
Chemokine expression in pNS1 or pNS2 transfected cells. A549 cells were transfected with pNS1 or pNS2 for 1 h and then treated with poly IC at a final concentration of 10 *μ*g/mL for the indicated times. The cultured supernatants were collected for determining the levels of MIP-*α* (a), RANTES (b), and IL-6 (c) using ELISA. The results are shown as the mean ± SE of three independent experiments.

Although the TLR3 and RIG-I proteins are both key sensors of viral dsRNA, our results indicate that the RSV NS protein induced SOCS1 upregulation independent of these factors. RSV infection induces chemokines such as RANTES, MIP-*α*, and IL-6 which are associated with childhood asthma and have side effects on type I IFN induction. Because of the potential roles of NS1 or NS2 on chemokines expression, we measured several TLR3-associated products that serve as triggers for inflammation. The concentrations of RANTES, MIP-*α*, and IL-6 were measured in the supernatants of cells transfected with pNS1 or pNS2 or treated with polyriboinosinic-polyribocytidylic acid (poly IC). Figures 5(a)–5(c) show that poly IC treatment elevated the levels of MIP-*α*, RANTES, and IL-6 at 24 h. However, the expressions of NS1 and NS2 obviously decreased the production of these poly IC-induced chemokines. This effect differed from RSV infection, which induces the production of chemokines. Thus, these NS proteins not only inhibited the IFN-inducible antiviral response by regulating SOCS feedback but also decreased chemokine induction.

## 4. Discussion

In this study, we conclude that the two RSV nonstructural proteins NS1 and NS2 interfere with the innate immune signaling through various steps: by upregulating SOCS1 and SOCS3 at an early phase and independent of autocrine IFN, by impairing type I IFN-inducible antiviral gene expression, and by impeding downstream signal transduction. In addition to these steps, NS1, NS2, and their combination decrease the expression levels of TLR3-dependent type II cytokines.

RSV* NS1* and* NS2*, which are located in the 3′ region of the viral genome, are essential triggers in inhibiting the host antiviral defense through the impairment of IFN-dependent antiviral responses [5, 35, 36]. The NS1 and NS2 proteins play different roles in downregulating IFN production by interfering with the combination of RIG-I with MAVS and by decreasing the IFN-related signaling pathway by degrading STAT2 [37]. The degradation of STAT2 by NS2 is likely to occur via a proteasomal mechanism [9, 38], whereas NS1 has the potential to act as an ubiquitin ligase enzyme (E3), targeting STAT2 to the proteasome. Both NS1 and NS2 are associated with inhibiting RIG-I-MAVS signaling. NS1 colocalizes with the mitochondrial antiviral signaling protein MAVS to inhibit the RIG-I-MAVS interaction required for IFN production [31], while NS2 antagonizes the activation of type I IFN transcription by interacting with RIG-I [27]. All of these data imply that NS proteins inhibit IFN antiviral signaling at multiple sites. Here, we present data showing that recombinant RSV NS proteins induce the expression of SOCS1 and SOCS3 in the early stages of transfection, leading to a functional inhibition of type I IFN-induced JAK/STAT phosphorylation. This provides an insight into the mechanism by which the RSV NS proteins suppress the host's antiviral defense and could ensure effective viral replication independent of the activation of endogenous type I IFN signaling.

In general, the binding of type I IFNs to a cognate receptor results in activation of the JAK/STAT pathway and the induction of SOCS family gene transcription levels in a STAT-dependent manner [39]. To establish a first line of defense against viral infection, the IFN response commences with the production of IFN-*α* and IFN-*β* in an autocrine and paracrine manner [40, 41]. NS1 mediated SOCS3 upregulation in the very early phase of transfection and was associated with higher levels of SOCS1 protein production, leading to a loss of STAT2 and STAT1/2 phosphorylation [33]. NS2 only contributed to the induction of SOCS1 expression but had a stronger inhibitory effect on STAT2 phosphorylation.

**Figure 6:**
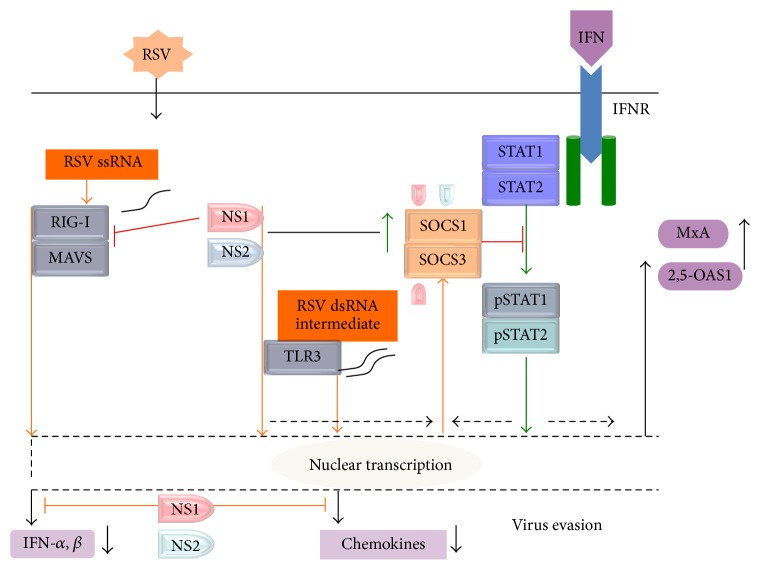
Model of RSV NS proteins activated SOCS1 and SOCS3 independent of endogenous IFN signaling. RSV replication and transcription releases NS1 and NS2 which upregulate SOCS1 and SOCS3 expression inhibit endogenous IFN-induced products and exogenous IFN-inducible signaling. NS1 induced SOCS1 and SOCS3 expression, while NS2 only induced SOCS1. Albeit both NS and type I IFN upregulate SOCS1 and SOCS3, NS suppressed the IFN-induced genes as well as the TLR3-dependent chemokines expression.

It has been demonstrated that influenza A virus induces SOCS1 and SOCS3 upregulation through a TLR3-independent, but RIG-I-MAVS-dependent, pathway [42]. Moreover, the overexpression of both SOCS1 and SOCS3 revealed that these factors strongly suppress the innate antiviral defense. These studies are further complemented by our findings that the RSV NS proteins are the major inducers of SOCS1 and SOCS3 upregulation but this mechanism involves neither a solely RIG-I-dependent nor a solely TLR3-dependent pathway. RSV NS1 binds to MAVS, inhibiting the RIG-I-MAVS interaction required for IFN production [43], while NS2 inhibits IFN activation at the level of RIG-I via a specific interaction that requires the 229 N-terminal amino acids of RIG-I [27]. Signals from* RIG-I* and* TLR3* might not be required for the production of SOCS proteins [44]. Usually, the transfer of a combined pSTAT1 and pSTAT2 heteromer drives SOCS gene transcription into the nucleus for promoter activation. There is a STAT-independent promoter that can activate SOCS nuclear transfection [44]. RSV NS1 is distributed to the nucleus and the cytoplasm to stimulate the production of HOXB5 and HOXB6 and thereby modulate nuclear transfer [37, 45]. Despite the significant upregulation of SOCS1 and SOCS3 by both viral NS proteins and IFN, they exert a different effect on the downstream products of antiviral gene expression. The IFN-induced physiological regulation of SOCS differs from that of the NS proteins, which inhibit IFN-inducible antiviral genes (Figure 6).

Many viruses disrupt the innate immune responses through the use of multifunctional viral proteins that target specific aspects of the NF-*κ*B pathway [46]. In addition, viral proteins are able to activate the I*κ*B kinase- (IKK-) related kinases, serine/threonine-protein kinase-1 (TBK-1), and IKK, both of which are known to be involved in the control of SOCS1 and SOCS3 expression [22, 23]. Previous studies have indicated that the* NS2* gene appeared to be more important in NF-*κ*B activation in RSV-infected cells than the* NS1* gene [7]. By contrast, in our study, transfection with pNS1 and pNS2 inhibited the production of chemokines, which implies that these viral proteins interfere with the TLR3-dependent NF-*κ*B pathway. One reason for this difference could be the fact that the artificially constituted plasmids used in this study are fusion proteins with flag and HA tags, which could affect the biological action of RSV NS proteins. There are limitations in this study; one is that we did not check how RSV NS proteins interact with nuclear transcription activation to promote SOCS1 and SOCS3 transcription and if there were other molecules participating in the modulation of this upregulation. Further study should be conducted to clarify the mechanism.

In summary, our results have demonstrated that RSV NS proteins exhibited a multifunctional capacity to impair the innate antiviral response through the early upregulation of SOCS expression. This effect was independent of cytokine triggering of the STAT phosphorylation pathway.
